# Minimally invasive surgical approach in children treated for oesophageal atresia is associated with attention problems at school age: a prospective cohort study

**DOI:** 10.1007/s00431-024-05449-y

**Published:** 2024-02-16

**Authors:** Anne-Fleur R. L. van Hal, John Vlot, Joost van Rosmalen, René M. H. Wijnen, Annabel P. J. M. van Gils-Frijters, Saskia J. Gischler, Lonneke M. Staals, Hanneke IJsselstijn, André B. Rietman

**Affiliations:** 1 Department of Paediatric Surgery, Erasmus Medical Centre Sophia Children’s Hospital, Rotterdam, the Netherlands; 2 Department of Biostatistics, Erasmus Medical Centre, Rotterdam, the Netherlands; 3 Department of Epidemiology, Erasmus Medical Centre, Rotterdam, the Netherlands; 4 Department of Child and Adolescent Psychiatry/Psychology, Erasmus Medical Centre Sophia Children’s Hospital, Rotterdam, the Netherlands; 5 Department of Anaesthesiology, Erasmus Medical Centre Sophia Children’s Hospital, Rotterdam, the Netherlands

**Keywords:** Anaesthetic exposure, Long-term follow up, Minimally invasive surgery, Neurocognitive outcome, Oesophageal atresia, Sustained attention

## Abstract

The long-term neurodevelopment of children born with oesophageal atresia (OA) is unclear. Therefore, we assessed the neurocognitive domains and their predictors. Patients born with OA between February 2006 and December 2014, who were routinely seen at eight years as part of a structured prospective longitudinal follow-up program, were included. Main outcome measures were need for school support, performance in various neurocognitive domains and potential predictors of neurocognitive problems. We analysed data of 65 children with a mean (SD) age of 8.1 (0.2) years, of whom 89% with OA type C. Thirty-five (54%) surgical corrections were minimally invasive; the median (interquartile range) duration of exposure to anaesthetics in the first 24 months was 398 (296 – 710) minutes. Forty-four (68%) attended regular education without extra support and intelligence was within normal range (99–108). More than 50% had z-scores ≤ -2 on one or more neurocognitive domains, of which attention was the most frequently affected domain. The speed on the sustained attention task was significantly below normal (z-score -1.48 (2.12), *p* < .001), as was fluctuation of sustained attention (z-score -3.19 (3.80), *p* < .001). The minimally invasive approach and a lower socio-economic status (both *p* = 0.006) proved significant predictors for sustained attention problems in multivariable analyses.

*  Conclusion*: Children who undergo minimally invasive surgery for OA correction are at risk for sustained attention problems at school age. Future studies unravelling the effects of perioperative events on neurodevelopment should lead to optimal surgical, anaesthesiological, and intensive care management in the neonatal period.

**Table:** 

**What is Known:** *• School-aged children born with oesophageal atresia have normal intelligence but problems with sustained attention at eight years.*	
**What is New:** *• Oesophageal atresia patients, who undergo minimally invasive surgery or who have a background of lower socioeconomic status are at serious risk for sustained attention problems at school age.* *• Moreover, those who have been intubated for a longer period are at risk for stronger fluctuations in sustained attention.*

**Supplementary Information:**

The online version contains supplementary material available at 10.1007/s00431-024-05449-y

## Introduction

Mortality and morbidity in children born with oesophageal atresia (OA) are primarily determined by major comorbidities and a complicated clinical course [1, 2]. Multiple surgeries and complications in the neonatal period necessitating prolonged hospitalisation affect development and daily functioning during childhood [3].

Neurodevelopment can be negatively affected by neonatal surgery and any acquired brain injury [4, 5]. A study in children with OA found impaired expressive language at preschool age [6]. Other studies found normal intelligence at school age but impaired attention and working memory [7, 8]. However, for the children born with OA, the potential causative factors behind these impairments have not yet been identified.

Minimally invasive surgery (MIS) provides better visualisation of the surgical field, with less surgical damage and better long-term outcome expectancy concerning scoliosis [9]. However, the effects of artificial CO_2_-pneumothorax to create a larger surgical workspace and the resulting hypercapnia and acidosis on the neonatal brain remain unclear. Moreover, this approach is technically more demanding than an open approach and often involves longer anaesthetic exposure [10–12].

After our previous assessment of neurocognitive functioning of school-aged children born with OA [7], we have introduced more extensive neurocognitive assessments since 2014 in our prospective, standardised follow-up program for children with congenital anatomical malformations [3]. Consistent with studies in survivors of neonatal extracorporeal membrane oxygenation and those with congenital diaphragmatic hernia [13], we hypothesised that school-aged children born with OA, who all have undergone interventions during the first years of life, are at risk for long-term neurocognitive problems that could potentially affect their school functioning. Hence, our objective was to assess the requirement for school support, evaluate performance in various neurocognitive domains, and identify predictors of neurocognitive problems.

## Method

### Population

We included data collected between April 2015 and June 2023 of 8-year-old children born with OA, seen in the context of the standardised prospective longitudinal follow-up program at the Erasmus MC – Sophia Children's Hospital [3].

Exclusion criteria were genetic syndromes known to affect neurodevelopment, leading to an inability to complete the neurocognitive test battery. The children's parents were informed that data were used for research purposes. This study was performed in line with the principles of the Declaration of Helsinki and was approved by the Medical Ethics Committee of the Erasmus University Medical Centre, the Netherlands (MEC-2017–185). Written informed consent was formally waived as there is no additional patient burden and no privacy concern. No funding has been received for this article.

### Data collection

Data from the medical records included: sex, gestational age (GA), prematurity (yes if GA < 37 weeks), birthweight, small for gestational age (SGA; i.e. birthweight < -2 SD) [14], type of OA according to Gross [15], congenital cardiac anomaly (yes, if surgery or follow-up by a cardiologist), VACTER-L association according to Solomon [16], surgical approach for OA correction, duration of anaesthetic exposure and number of procedures under general anaesthesia within the first 24 months, need for tracheotomy or gastrostomy, duration of intubation (pre- and postoperative, including re-intubation), sepsis during first hospital admission (positive blood culture), duration of initial hospital stay, feeding type at discharge, history of fundoplication surgery.

Additionally, we categorised type of education (regular, regular with extra help or special education), and socio-economic status (SES) according to highest maternal education level (International Standard Classification of Education (ISCED) levels low (0–4) and high (5–8) [17].

#### Neurocognitive assessment

An experienced paediatric psychologist performed the following assessments in the outpatient clinic (see Supplemental File 1 for details):

1. Intelligence Quotient (IQ)
  - Twelve or ten subtests of the Wechsler Intelligence Scale for Children (respectively WISC-III-NL or WISC-V-NL) to assess Verbal Comprehension (VC), Perceptual Organisation (PO), Processing Speed (PS) and Total IQ (TIQ) [18].

For organisational reasons, seven children born in 2006/2007 were subjected to only five subtests [18].

2. Attention
  - Processing speed: Trail Making Test, section A (TMT-A)
  - Selective attention and cognitive flexibility: Stroop colour-word test (Stroop)
  - Sustained attention: Dot Cancellation Test with series time (DCT-Time) and standard deviation of the series time (DCT- SD), representing the fluctuation of attention.
3. Verbal-memory
  - Verbal working memory: Digit Span subtest of the WISC-tests
  - Immediate and delayed recall: Rey Auditory Verbal Learning Test (RAVLT)
4. Visuospatial processing
  - Copy of the Rey Complex Figure Test (RCFT Copy)
5. Visuospatial Memory
  - Working memory: Spatial Span subtest of the Wechsler Nonverbal Scale of Ability (WNV)
  - Immediate and delayed recall: Rey Complex Figure Test (RCFT)
6. Executive functioning
  - Cognitive flexibility: Trail Making Test, section B (TMT-B)
  - Strategy/planning: Key Search and Modified Six Elements of the Behavioural Assessment of the Dysexecutive Syndrome (BADS-C-NL)
  - Parent-rated daily executive functioning: Behaviour Rating Inventory of Executive Function (BRIEF)

Neurocognitive test scores were converted into z-scores and – if applicable – inverted so that a higher score always equals better performance. Z-scores ≤ -2 were regarded as reflecting impaired functioning; z-scores > -2 and ≤ -1 were regarded as reflecting borderline functioning (general population: mean z-score = 0; SD = 1) [19].

### Statistical analysis

The Shapiro–Wilk Test was used to assess normality of continuous variables. Parametric tests were used to evaluate differences in normally distributed continuous patient characteristics between participating and non-participating patients (independent samples t-test) and to assess whether the normally distributed neurocognitive test outcomes (one-sample t-test) differed from population norms. Non-parametric tests were used for categorical (Fisher’s exact test) and non-normally distributed patient characteristics (Mann–Whitney U test) and neurocognitive test outcomes (one-sample Wilcoxon signed-rank test). All data are shown as mean (SD), median (interquartile range (IQR)), or proportions (%). The children who did not attend the follow-up program and those who had not undergone assessment of intelligence with WISC-III or V, were considered as non-participants.

To evaluate the predictor variables for impaired neurocognitive outcome, we conducted univariable and multivariable linear regression analyses for the outcome measures that scored significantly below the norm. Based on clinical experience and earlier studies, we chose five predictor variables, reflecting background (GA and SES), initial treatment (MIS (yes/no) and duration of intubation), and clinical course (duration of anaesthetic exposure). The duration of intubation was coded as a categorical variable with categories 1–2 days and ≥ 3 days. The predictor variables were checked for outliers. The multivariable linear regression included all five predictor variables. The amount of and reasons for missing data were evaluated. In case data were considered missing at random, multiple imputation of missing values was performed using a fully conditional specification [20]. The missing values for type of surgical approach were however not imputed, as we did not consider this appropriate. Patients with unknown surgical approach were not included in multivariable analysis. Thirty imputed data sets were generated for each outcome, and the results were pooled using Rubin’s rules. The assumptions for linear regression analysis were assessed using normal probability plots of the residuals and by calculating variance inflation factors. Multicollinearity was assumed if variance inflation factors exceeded 2.5 [21].

Analyses were performed with SPSS 25.0 (IBM, Armonk, NY, USA), and a two-sided p-value < 0.05 was considered statistically significant. Due to the study's explorative nature, correction for multiple comparisons was not performed.

## Results

### Patient characteristics

**Fig. 1:**
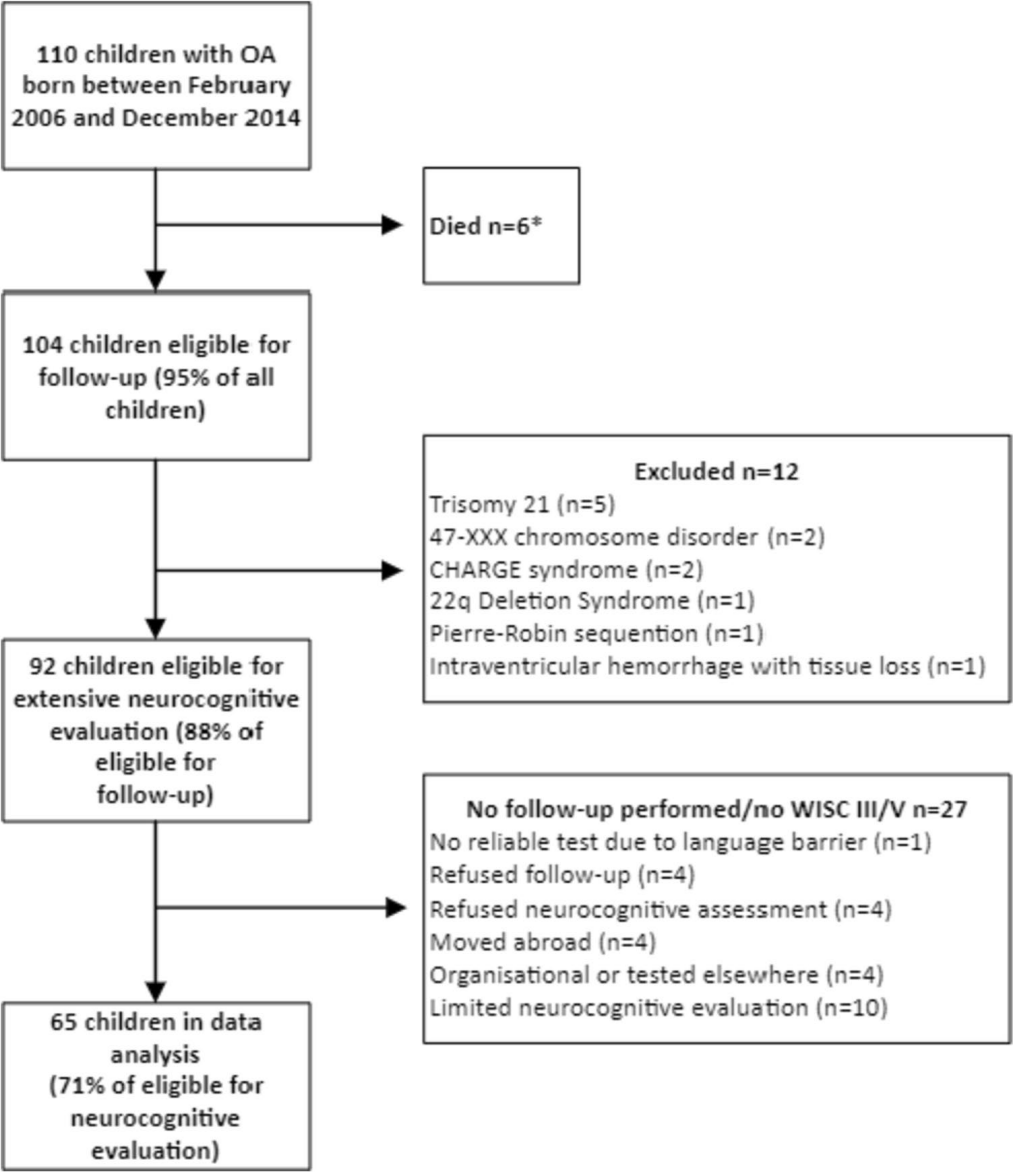
Study inclusion flowchart. OA = oesophageal atresia. *Trisomy 18, Fanconi anaemia with complicated neonatal course, refractory epilepsy with infaust prognosis, triventricular hydrocephalus post-resuscitation (all N = 1), multiple major congenital anomalies (N = 2).

Of 110 children born with OA between February 2006 and December 2014, six had died before the age of eight, and twelve were excluded based on genetic syndromes or psychomotor retardation. Eventually, data from 65 children were analysed (Fig. 1). Their mean age was 8.1 (0.2) years. The baseline characteristics of participants and non-participants did not significantly differ (Table 1).

**Table 1:** Characteristics of participating (N = 65) and non-participating children (N = 27)

	**Participants**	**Non-participants**	***p*-value**
**Sociodemographic characteristics**			
Sex			0.085^a^
Girls	22 (34)	14 (52)	
Boys	43 (66)	13 (48)	
Socio-economic status			0.625^a^
Low	28 (43)	13 (48)	
High	29 (45)	10 (37)	
Missing	8 (12)	4 (15)	
**Clinical data**			
Gestational age (weeks)	38 (36–39)	38 (36 – 40)	0.793^c^
Prematurity (< 37 weeks)	27 (42)	10 (37)	0.816^a^
Birthweight (g)	2788 (2000 – 3120)	2668 (2050 – 3063)	0.688^b^
Small for gestational age	30 (46)	10 (37)	0.484^a^
Type of oesophageal atresia			0.696^a^
Gross type A (isolated OA)	3 (5)	2 (7)	
Gross type B (OA with proximal TEF)	3 (5)	0	
Gross type C (OA with distal TEF)	58 (89)	25 (93)	
Gross type D (OA with dual TEF)	1 (2)	0	
Congenital cardiac anomaly*	5 (8)	0	0.317^a^
VACTER-L	11 (17)	8 (30)	0.161^a^
Surgical approach for OA correction			0.411^a^
Minimally invasive surgery	35 (54)	12 (44)	
Thoracotomy Converted	27 (42) 2 (3)	15 (56) 0	
Duration of anaesthetic exposure within the first 24 months of life, minutes	398 (296 – 710)	439 (304 – 783)	0.630^c^
General anaesthetic procedures within the first 24 months of life	3 (2 – 6)	4 (1 – 7)	0.573^c^
Need for tracheotomy	1 (2)	1 (4)	0.501^a^
Need for gastrostomy	12 (18)	7 (26)	0.404^a^
Duration of intubation**, days	2 (1 – 3)	2 (1 – 6)	0.138^c^
Sepsis during first hospital admission	3 (5)	4 (15)	0.187^a^
Duration of initial hospital stay, days	20 (12 – 35)	21 (12 – 66)	0.691^c^
Feeding type at initial discharge			0.556^a^
No tube feeding	41 (63)	13 (48)	
(Supplemental) tube feeding	13 (20)	7 (26)	
Gastrostomy	8 (12)	5 (19)	
Missing	3 (5)	2 (7)	
Fundoplication surgery	12 (18)	7 (26)	0.573^a^
Age at fundoplication surgery (days)	124 (63 – 250)	143 (66 – 198)	0.957^c^

Data are expressed as median (IQR) or number (percentage), as appropriate

*OA* oesophageal atresia, *TEF* tracheoesophageal fistula, *IQR* interquartile range

^a^Fisher's exact test

^b^Independent samples t-test

^c^Mann-Whitney U test

*Congenital cardiac anomalies requiring surgery: ventricular septal defect (VSD) (N=1), total anomalous pulmonary venous return and closure atrial septal defect (N=1), atrial septal defect (N=1), Tetralogy of Fallot (N=2)

**Five outliers in the duration of intubation, reasons: perinatal asphyxia (9 days; N = 1), total anomalous pulmonary venous return and pulmonary hypoplasia (94 days; N = 1), oesophageal atresia-related pulmonary complications (27 days, 37 days and 45 days; N = 3)

Fifty-eight participants had Gross type C. The overall median gestational age was 38 (IQR 36–39) weeks. Twenty-seven children were born prematurely, with a range from 29 up to 37 weeks. In 37 cases (57%), the primary OA correction was minimally invasive, with two conversions to open surgery. Minimally invasive surgery was performed when the patient was cardiopulmonary-stable and when deemed surgically feasible. The median duration of exposure to anaesthetics within the first 24 months was 398 (IQR 269–710) minutes in 3 (IQR 2–6) surgical and/or diagnostic procedures under anaesthesia. The median duration of intubation was 2 (IQR 1–3) days, and the initial hospital stay was 20 (IQR 12–35) days.

For GA, two missing values were imputed, for anaesthetic exposure twelve, for duration of intubation ten and for SES eight patients. Missing data for these variables were due to initial treatment in other centres.

### Neurocognitive outcome

Forty-four children (68%) attended regular education without extra support. Seventeen children (26%) received support to keep up with regular education. Four children attended special education (6%). This distribution is close to the general Dutch population's [22].

**Fig. 2:**
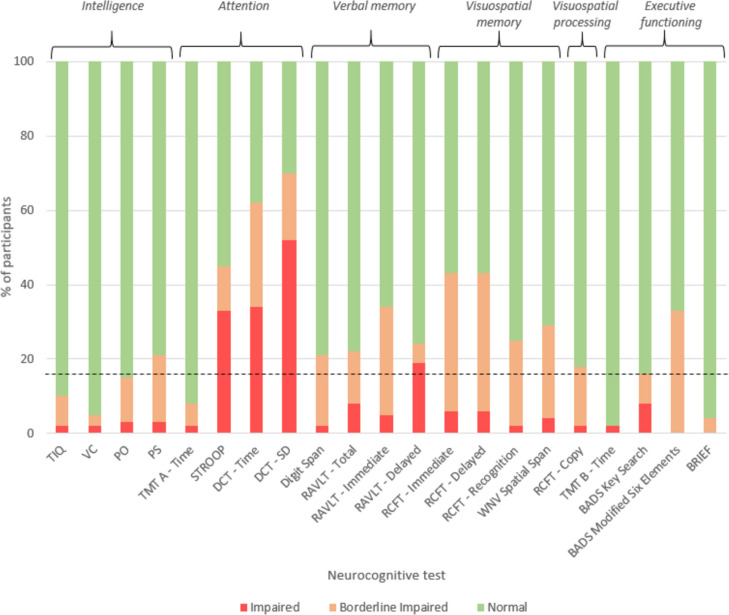
Distribution of test scores in the study population. Red: percentage of patients with a z-score ≤ -2 (impaired), orange: -2 ≤ -1 (borderline), and green: > -1 (normal) on each of the neurocognitive tests. The dotted line at 16% of the population represents the expected proportion of children having z-scores ≤ -1. Abbreviations: TIQ = Total IQ; VC = Verbal Comprehension; PO = Perceptual Organisation; PS = Processing Speed; TMT = trial making test; Stroop = Stroop colour word test; DCT = dot cancellation test; RAVLT = Rey auditory verbal learning test; RCFT = Rey complex figure test; WNV = Wechsler Nonverbal Scale of Ability; BADS = Behavioural Assessment of the Dysexecutive Syndrome; BRIEF= Behaviour Rating Inventory of Executive Function.Predictors of neurocognitive outcome

The total IQ and verbal comprehension were above general population norms (Table 2; Fig. 2). The parents rated the daily executive functioning favourably (Table 2).

**Table 2:** Overview of neurocognitive outcome compared to norm scores

Neurocognitive test	N	Outcome	*p*-value
*Intelligence*			
** WISC-III-NL/WISC-V-NL**			
Total IQ (TIQ)	65	105 (15)	**0.017**
Verbal Comprehension (VC)	57	108 (14)	** < 0.001**
Perceptual Organisation (PO)	56	99 (15)	0.669
Processing Speed (PS)	64	101 (15)	0.765
*Attention*			
* Selective attention*			
** TMT A** Time z-score	63	0.55 (0.87)	** < 0.001**
** Stroop**			
Interference z-score	58	-1.25 (2.97	**0.007**
* Sustained attention*			
** DCT**			
Series time z-score	61	-1.48 (2.12)	** < 0.001**
SD series z-score	61	-3.19 (3.80	** < 0.001**
*Verbal memory*			
** WISC-III-NL/WISC-V-NL Digit Span**			
Total z-score	64	-0.16 (0.81)	0.126
** RAVLT**			
Total z-score	63	-0.21 (1.09)	0.136
Immediate z-score	63	-0.27 (1.18)	0.077
Delayed z-score	59	-0.43 (1.41)	0.151
*Visuospatial memory*			
** RCFT immediate**			
Immediate z-score	62	-0.50 (1.17)	**0.002**
** RCFT delayed**			
Delayed z-score	62	-0.45 (1.13)	**0.003**
** RCFT recognition**			
Recognition z-score	62	-0.03 (0.95)	0.832
*Visuospatial working memory*			
** WNV Spatial Span**			
Spatial Orientation z-score	52	-0.28 (1.01)	**0.047**
*Visuospatial processing*			
** RCFT copy**			
Copy z-score	62	0.13 (0.91)	0.252
*Executive functioning*			
* Cognitive flexibility*			
** TMT-B**			
Time z-score	58	0.38 (0.74)	** < 0.001**
* Strategy formation*			
** Key Search**			
z-score	63	0.23 (1.10)	0.313
* Planning*			
** Modified Six Elements**			
z-score	42*	-0.50 (0.81)	** < 0.001**
*Parent-rated daily executive functioning*			
**Parent Proxy Report**	54	0.69 (1.13)	** < 0.001**
BRIEF total z-score			

Mean (standard deviation) = average IQ score or z-score of neurocognitive test. Compared to the norm population with a population IQ mean of 100 (SD = 15) or mean z-score of 0 (SD = 1). One-sample t-test was used for normally distributed data, and one-sample Wilcoxon signed-rank test for not normally distributed data

*WISC-III-NL* Wechsler Intelligence Scale for Children, third, Dutch version, *TIQ* total IQ, *VC* verbal comprehension, *PO* perceptual organisation, *PS* processing speed, *TMT* trial making test, *Stroop* Stroop colour word test, *DCT* dot cancellation test, *SD* standard deviation, *RAVLT* Rey auditory verbal learning test, *RCFT* Rey complex figure test, *WNV* *Spatial Span* Wechsler Nonverbal Scale of Ability, subtest Spatial Span, *BRIEF* Behaviour Rating Inventory of Executive Functioning (see Supplemental File 1 for neurocognitive test descriptions)

*Missing data due to hygiene protocol during the COVID-19 pandemic

In the neuropsychological assessment, the scores for short- and long-term visuospatial recall (RCFT-immediate and delayed), visuospatial working memory (WNV Spatial Span), and executive functioning subdomain planning (Modified Six Elements) were significantly below normal (Table 2).

Attention was the only domain with mean z-score < -1, more specifically the subdomain of sustained attention. The DCT showed a borderline impaired speed (series time z-score -1.48 (2.12); *p* < 0.001) and impaired fluctuation (series SD z-score -3.19 (3.80); *p* < 0.001). The Stroop indicated borderline impaired selective attention and cognitive flexibility (interference z-score -1.25 (2.97); *p* = 0.007).

Figure 2 shows performance in different neurocognitive domains. Over 50% of the children had z-scores ≤ -2 on one or more tests (Fig. 2). For fourteen tests, the proportion of children with results in the borderline range was higher than expected. For twelve tests – three assessing attention – the proportion of children with results in the impaired range was higher than expected.

In univariable regressions, lower SES was associated with impaired sustained attention (*p* = 0.034), increased fluctuation of sustained attention (*p* = 0.038), impaired visuospatial working memory (*p* < 0.001), and impaired delayed visuospatial memory (*p* = 0.010) (Table 3; Supplemental File 2). In addition, a longer duration of intubation was significantly associated with more fluctuation of sustained attention (*p* = 0.030).

**Table 3:** Univariable and multivariable linear regression analyses with predefined variables for the DCT – Time, DCT – SD and WNV Spatial Span

	Univariable linear regression analyses			Multivariable linear regression analyses		
	B*	95% CI	*p*-value	B*	95% CI	*p*-value
*Potential predictors for impaired ****sustained attention***						
Gestational age (weeks)	-0.049	-0.546 – 0.448	0.843	0.188	-0.342 – 0.718	0.488
Surgical approach^a^	-2.630	-5.367 – 0.106	0.059	-3.713	-6.335 – -1.092	**0.006**
Duration of anaesthetic exposure (minutes)	0.003	-0.001 – 0.006	0.127	0.004	-1.737E-5 – 0.009	0.051
Duration of intubation^b^	1.904	-1.412 – 5.219	0.254	1.145	-1.829 – 4.119	0.450
SES ^c^	-2.994	-5.751 – -0.236	**0.034**	-3.752	-6.400 – -1.104	**0.006**
*Potential predictors for increased ****fluctuation of sustained attention***						
Gestational age (weeks)	-0.182	-0.407 – 0.044	0.112	-0.105	-0.344 – 0.134	0.389
Surgical approach^a^	-1.019	-2.292 – 0.253	0.114	-1.598	-2.781 – -0.415	**0.008**
Duration of anaesthetic exposure (minutes)	0.001	0.000 – 0.003	0.087	0.001	-0.001 – 0.003	0.275
Duration of intubation ^b^	1.633	0.168 – 3.098	**0.030**	1.142	-0.175 – 2.458	0.089
SES ^c^	-1.319	-2.562 – -0.075	**0.038**	-1.595	-2.766 – -0.425	**0.008**
*Potential predictors for impaired ****visuospatial working memory***						
Gestational age (weeks)	0.160	-0.197 – 0.518	0.372	0.038	-0.066 – 0.142	0.471
Surgical approach^a^	1.074	-0.714 – 2.862	0.233	0.483	-0.049 – 1.016	0.075
Duration of anaesthetic exposure (minutes)	-0.001	-0.004 – 0.003	0.732	-4.463E-5	-0.001 – 0.001	0.931
Duration of intubation ^b^	-1.605	-3.637 – 0.427	0.118	-0.418	-1.025 – 0.190	0.177
SES ^c^	3.007	1.381 – 4.633	** < 0.001**	0.921	0.426 – 1.415	** < 0.001**

DCT-Time representing sustained attention, DCT-SD the fluctuation of sustained attention and WNV Spatial Span the visuospatial working memory

*DCT* dot cancellation test, *SD* standard deviation, *WNV* Wechsler Nonverbal Scale of Ability, *SES* socio-economic status

^a^0 = minimally invasive surgery/converted and 1 = thoracotomy

^b^0 = 1–2 days and 1 =  ≥ 3 days

^c^0 = low and 1 = high

*Unstandardized Beta

In multivariable regression analyses with multiple imputation to account for data missing at random, MIS and lower SES (both *p* = 0.006) were associated with sustained attention problems (Table 3). The MIS and lower SES were also significantly associated with sustained attention fluctuation (both *p* = 0.008) (Table 3). The remaining univariable and multivariable regression analyses are provided in Supplemental File 2. All variance inflation factors were below 2.5, thus no multicollinearity was assumed.

## Discussion

This study is – to our knowledge – the first to extensively evaluate multiple domains of neurocognitive outcome and its association with MIS and other potential predictors in school-aged children born with oesophageal atresia. We showed that these children had normal school performance and intelligence within normal ranges. Still, sustained attention problems were noted, suggesting that school-aged children with OA can focus briefly on a task but do not sustain attention easily. Significant independent predictors for impaired sustained attention and its fluctuation were MIS and a background of lower SES. Test scores slightly below the norm are mainly within the neurocognitive domains (verbal and visuospatial memory) where attention is required for adequate performance. In univariable analyses, lower SES and longer duration of intubation were predictors of sustained attention fluctuation. However, in multivariable analysis, the duration of intubation did not remain a significant predictor. Children with low SES are also at risk for impaired visuospatial working memory and delayed visuospatial memory.

Several previous studies on neurocognitive outcomes in children born with OA showed impairments throughout different stages of childhood. Burnett and co-workers longitudinally studied the cognitive, academic, and behavioural functioning of 71 five-year-old and 72 eight-year-old OA patients [8]. The children were at risk for cognitive difficulties, particularly in attention and working memory, not significantly associated with additional congenital anomalies, duration of hospitalisation, or prematurity [8]. Earlier, we demonstrated sustained attention problems at school age in a cohort of OA children born between 1999 and 2006; before introducing the minimally invasive approach in our centre [7]. Two studies reported a higher prevalence of intellectual disabilities with special educational needs than we did: in 22% of school-aged OA children and 33% of adolescents, respectively [23, 24]. A Swedish national registry-based study showed a higher risk of autism spectrum disorders and intellectual disability in 735 adults born with OA [25]. Other than this Swedish study, no data on more profound psychiatric diagnostics has been published to date.

Previous studies have not identified possible predictors for impaired neurocognitive outcomes in individuals born with OA. However, in the broader perspective of non-cardiac congenital anomalies, multiple risk factors for developmental delay in infants have been identified, such as longer duration of mechanical ventilation, parental education level [4], and undergoing numerous surgical interventions in the first 24 months [3, 4].

In this study, MIS was negatively associated with the speed and fluctuation of sustained attention. MIS is technically more demanding and generally takes longer to complete than the open approach [10–12]. Moreover, the artificial CO_2_-pneumothorax used to create surgical workspace results in a significantly higher pCO_2_ level in the patient [26, 27]. This additional CO_2_ load may result in hypercapnia and acidosis [28, 29], the effects of which on the neonatal brain are still unclear. This study suggests a possible association between MIS and neurocognitive outcomes at school age. However, the cohort examined in this study underwent surgery 8–17 years ago. Since then, surgeons and anaesthesiologists have gained more experience with the surgical and anaesthesiological aspects of MIS, and the techniques have been further developed. Therefore, future research with new cohorts is needed to investigate whether single or multiple surgeries using these techniques affect the neonatal brain.

The reduced sustained attention should be seen in the context of this cohort's relatively high intelligence scores. The high intelligence in our cohort could be associated with the high SES [30]. Concomitant with the high intelligence scores, at least average attention scores would be anticipated. This further emphasises the discrepancy between intelligence and impaired attention in our cohort. Therefore, parents of children with attentional issues need counselling at school age [31]. In addition, the current study must be considered when deliberating treatment options for these patients, both in infancy and school age.

Our study – which contributes to knowledge on neurocognition and potential risk factors for adverse outcomes in school-aged children with OA – underlines the importance of previously advocated standardised follow-up programs for these children [8, 32, 33]. Further research into predictors of neurocognitive outcomes is critical to improve care for individuals born with OA [7, 8, 24, 32, 33].

Strengths of our study are the relatively large cohort, who prospectively attended the same structured follow-up program, and the high participation rate (71% of eligible candidates). We found no evidence of selection bias, with similarity in baseline characteristics in participants and non-participants. Still, some potential drawbacks associated with our study should be mentioned. The duration of anaesthetic exposure was considered as one of the predictors, but we did not account for the choice of anaesthetics and the occurrence of perioperative respiratory or hemodynamic events. Unfortunately, our medical records’ data did not permit such detailed analyses.

## Conclusion

We demonstrated that children born with OA are at risk for sustained attention problems at school age, highlighting the need for counselling and timely guidance in educational settings. MIS was identified as an important independent risk factor for sustained attention problems. Future research should employ multimodal neuromonitoring with larger sample sizes and include more detailed perioperative and anaesthesiological parameters linked to neurodevelopmental outcomes. This will enable a better understanding and guide both surgeons and anaesthesiologists in their choices regarding treatment.

### Supplementary Information

Below is the link to the electronic supplementary material.

Supplementary file1 (DOCX 27 KB)

Supplementary file2 (DOCX 19 KB)

## Data Availability

No datasets were generated or analysed during the current study.

## Abbreviations

BADS-C-NL: Behavioral Assessment of the Dysexecutive Syndrome Dutch version
BRIEF: Behavior Rating Inventory of Executive Function
DCT: Dot Cancellation Test
GA: Gestational Age
ISCED: International Standard Classification of Education
IQ: Intelligence Quotient
MICE: Multivariate Imputation by Chained Equations
MIS: Minimally Invasive Surgery
OA: Oesophageal Atresia
PO: Perceptual Organization
PS: Processing Speed
RAVLT: Rey Auditory Verbal Learning Test
RCFT: Rey Complex Figure Test
SD: Standard Deviation
SES: Socio-economic Status
SGA: Small for Gestational Age
TEF: Tracheoesophageal Fistula
TIQ: Total Intelligence Quotient
TMT: Trail Making Test
VC: Verbal Comprehension
VSD: Ventricular Septal Defect
WISC-III-NL: Wechsler Intelligence Scale for Children, third, Dutch version
WISC-V-NL: Wechsler Intelligence Scale for Children, fifth, Dutch version
