# Supplementary material for: Minimally invasive surgical approach in children treated for oesophageal atresia is associated with attention problems at school age: a prospective cohort study
Source: Eur J Pediatr. 2024 Feb 16;183(5):2131–40. doi: 10.1007/s00431-024-05449-y (PMC11035457; doi:10.1007/s00431-024-05449-y)
Supplement: Supplementary file 1 — Supplementary file1 (DOCX 27 KB) [file 431_2024_5449_MOESM1_ESM.docx]

**Supplemental File 1. Descriptions of the neurocognitive tests^*^**

Validated neurocognitive tests were administered in their Dutch standardised versions to assess skills in six domains as described previously (1).

*Intelligence*

Wechsler Intelligence Scale for Children (WISC-III-NL)

The Wechsler Intelligence Scales for Children (WISC-III-NL 2014- 2020 and WISC-V-NL 2021- 2023) were used. Both tests assess verbal and non-verbal intelligence, have been shown to have good reliability and validity (2, 3), and have been used by our group before (4, 5). A normalized population mean of 100 for both tests with a standard deviation of 15 is applicable (2, 3).

The shortened IQ assessment used for children born between April 2006 and May 2007 (N=7) consisted of five subtests: Block Design, Vocabulary, Coding, Symbol Search, and Digit Span. The IQ was calculated based on only Block Design, Vocabulary.

The majority of the children were assessed using the WISC-III-NL, therefore Verbal Comprehension, Perceptual Organisation, and Processing Speed were used for the intelligence assessment. Although the content of the scales differs, corresponding scales from the WISC-V-NL and the WISC-III-NL were used as much as possible. For the Verbal Comprehension, the score Verbal Comprehension Index was used, for Perceptual Organisation the Visual Spatial Index score, and for the Processing Speed, the Processing Speed Index score.

*Attention*

Trail Making Test (TMT)

This paper and pencil test consists of two parts. In the first part (part A), the subject must draw lines to consecutively connect numbered circles on a sheet. In the second part (part B), the subject must consecutively but alternately connect numbered and lettered circles on another worksheet. The goal of the test is to finish each part as quickly as possible. The test can be administered to children and adults in the age range of 6-89 years. This test measures visual conceptual and visuomotor tracking (part A) as well as cognitive flexibility (part B) (5, 6).

Stroop Colour Word Test (Stroop)
The Stroop consists of three trials: in the first trial (Stroop 1) the subject must read names of colours, in the second trial (Stroop 2) name colours of coloured blocks, and in the third trial (Stroop 3) name printed colours without being distracted by the colour name. The test can be administered to children and adults in the age range of 8-65 years. Selective attention is measured with this test (5, 6).

Dot Cancellation Test (DCT; Bourdon-Vos test)

This paper-and-pencil test measures sustained selective attention in terms of speed, variability of speed, and accuracy. It consists of a paper on which figures made of three, four or five dots are displayed in 33 rows. The child is instructed to cross off all figures with four dots, as precise and as fast as they can (7).

*Verbal memory*

WISC-III-NL – subtest Digit Span and WISC-V-NL – Digit Span scaled score

The Digit Span subtest consists of random number sequences that increase in length which the examiner reads aloud at the rate of 1 number per second. The child has to reproduce these numbers in the same order. Next, the sequences must be recalled backwards (3-5-7 becomes 7-5-3). The first part of the test measures short-term auditory memory and short-term retention capacity. The second part measures auditory working memory (8). In the WISC-V, an additional third part asks the child to put numbers in the right order before reproducing them.

Rey Auditory Verbal Learning Test (RAVLT)

The RAVLT consists of five presentations with a recall of a 15-word list (Immediate recall), and a sixth recall trial after 30 minutes (Delayed recall). This test measures verbal memory span, short- and long-term verbal memory, and verbal recognition. It can be administered to children and adults in the age range of 6-89 years (9, 10).

*Visuospatial memory*

Rey Complex Figure Test (RCFT)

The RCFT consist of three trials. First, the child has to copy a complex figure (Copy). This part measures visuospatial processing. Then after 3 minutes (Immediate recall) and after 30 minutes (Delayed recall) the figure must be drawn from memory. Next, different figures are shown and the child has to indicate whether these figures were in the original figure (Recognition). The last two trials measure short- and long-term visual-spatial memory and visual-spatial recognition. This test can be completed by children and adults in the age range of 6-89 years (11, 5).

Wechsler Nonverbal Scale of Ability (WNV) – subtest Spatial Span

Spatial Span requires the child to touch a group of blocks arranged on a board in a non-systematic manner in the same and reverse order as demonstrated by the examiner. The first part of the test measures short-term visuospatial memory and short-term retention capacity. The second part measures visuospatial working memory (12).

*Executive functioning*

Two tasks are taken from the BADS-C (Behavioural Assessment of the Dysexecutive Syndrome for Children):

Key Search

A test of strategy formation. The child is asked to demonstrate how they would search a field for a set of lost keys and their strategy is scored according to its functionality (13).

Modified Six Elements
The child is asked to work on six different tasks for which they have five minutes. There are some rules the child has to obey during the task while making sure that by the end of the five minutes, parts of all six of the tasks have been done and the child has done as much as possible of each task. This is a test of planning, task scheduling and performance monitoring (13).

*Parent-rated daily executive functioning*

Behaviour Rating Inventory of Executive Functioning (BRIEF)

The BRIEF is a parent-completed questionnaire that provides data on the extent to which executive dysfunction impairs a child in his or her natural settings, for children and adolescents aged 5-18 (14). The questionnaire takes approximately 10-15 minutes and covers eight domains: inhibition, shifting, emotional control, initiation, working memory, planning/organization, organization of materials and monitoring. These subtests are used to construct total scores: Behavioural Regulation (three scales), Metacognition (five scales), and a Global Executive Composite (all scales).

**REFERENCES**

1. Leeuwen L, Schiller RM, Rietman AB, van Rosmalen J, Wildschut ED, Houmes RJM, et al. Risk Factors of Impaired Neuropsychologic Outcome in School-Aged Survivors of Neonatal Critical Illness. Crit Care Med. 2018;46(3):401-10.

2. Bleichrodt N, Drenth PJD, Zaal JM, Resing WCM. Intelligentiemeting bij Kinderen (Intelligence testing in children). Lisse: Zwets en Zeitlinger; 1987.

3. Kort W, Compaan EL. WISC NL III. Handleiding. NIP Dienstencentrum 1999.

4. Madderom MJ, Toussaint L, van der Cammen-van Zijp MH, Gischler SJ, Wijnen RM, Tibboel D, IJsselstijn H. Congenital diaphragmatic hernia with(out) ECMO: impaired development at 8 years. *Arch Dis Child Fetal Neonatal Ed* 2013; 98: F316-322.

5. Lezak MD, Howieson DB, Loring DW. Neuropsychological assessment, 4th ed. Oxford: Oxford University Press; 2004.

6. Schmand B, Houx P, De Koning I. Dutch norms for Stroop colour-word test, Trail making test, Rey auditory verbal-learning test, Verbal fluency, and Story recall of Rivermead behavioural memory test. Amsterdam: Division Neuropsychology of the Dutch Institute for Psychology; 2003.

7. Vos P. Bourdon-Vos. Handleiding (manual dot cancellation test). Lisse: Swets en Zeitlinger; 1992.

8. Uterwijk J. WAIS-III Dutch Technical Manual. Amsterdam: Pearson Test Publisher; 2000.

9. van den Burg W, Kingma A. Performance of 225 Dutch school children on Rey's Auditory Verbal Learning Test (AVLT): parallel test-retest reliabilities with an interval of 3 months and normative data. *Arch Clin Neuropsychol* 1999; 14: 545-559.

10. Schmidt M. Rey Auditory and Verbal Learning Test: a handbook. Los Angeles, CA: Western Psychological Services; 1996.

11. Meyers JE, Meyers, K.R. Rey Complex Figure Test and Recognition Trial Supplemental Norms for Children and Adolescents. Lutz: Psychological Assessment Resources; 1996.

12. Wechsler D, Naglieri JA. Wechsler Nonverbal Scale of Ability San Antonio, TX: Pearson; 2006.

13. Emslie H, Wilson FC, Burden V, Nimmo-Smith I, Wilson BA. Behavioral assessment of the dysexecutive syndrome for children (BADS-C), Dutch version. Amsterdam Harcourt; 2006.

14. Gioia, G.; Isquith, P.K.; Guy, S.C.; Kenworthy, L. (2000). Reviewed by Baron, I.S. "Test Review: Behavior Rating Inventory of Executive Function". *Child Neuropsychology*. **6** (3): 235–238. [doi](https://en.wikipedia.org/wiki/Doi_(identifier)):[10.1076/chin.6.3.235.3152](https://doi.org/10.1076%2Fchin.6.3.235.3152).
