# Supplementary material for: Minimally invasive surgical approach in children treated for oesophageal atresia is associated with attention problems at school age: a prospective cohort study
Source: Eur J Pediatr. 2024 Feb 16;183(5):2131–40. doi: 10.1007/s00431-024-05449-y (PMC11035457; doi:10.1007/s00431-024-05449-y)
Supplement: Supplementary file 2 — Supplementary file2 (DOCX 19 KB) [file 431_2024_5449_MOESM2_ESM.docx]

**Supplemental File 2. Univariable and multivariable linear regression analyses with predefined variables for the Stroop, RCFT – Immediate, RCFT – Delayed and BADS-C-NL Modified Six Elements.**

**Table X.** Univariable and multivariable linear regression with predefined variables for *selective attention and cognitive flexibility* (Stroop), *immediate visuospatial memory* (RCFT – Immediate), *delayed visuospatial memory* (RCFT – Delayed) and *planning* (BADS-C-Modified Six Elements). ^α^0 = minimally invasive surgery/converted and 1 = thoracotomy; ^β^0 = 1-2 days and 1 = ≥3 days; ^γ^0 = low and 1 = high. Abbreviations: RCFT = Rey Complex Figure Test; BADS-C= Behavioural Assessment of the Dysexecutive Syndrome for Children; SES = socio-economic status. *Unstandardized Beta.

|  | Univariable linear regression analyses | | | Multivariable linear regression analyses | | |
| --- | --- | --- | --- | --- | --- | --- |
|  | B* | 95% CI | *p*-value | B* | 95% CI | *p*-value |
| *Potential predictors for impaired* ***selective attention and cognitive flexibility*** | | | | | | |
| Gestational age (weeks) | -0.013 | -0.310 – 0.283 | 0.928 | -0.002 | -0.358 – 0.355 | 0.993 |
| Surgical approach ^α^ | 0.874 | -0.747 – 2.495 | 0.285 | 1.163 | -0.487 – 2.813 | 0.167 |
| Duration of anaesthetic exposure (minutes) | -5.261E-6 | -0.002 – 0.002 | 0.996 | 0.000 | -0.002 – 0.003 | 0.808 |
| Duration of intubation ^β^ | -1.177 | -3.139 – 0.784 | 0.233 | -1.291 | -3.257 – 0.674 | 0.198 |
| SES ^γ^ | 1.339 | -0.359 – 3.037 | 0.120 | 1.542 | -0.104 – 3.189 | 0.066 |
| *Potential predictors for impaired* ***immediate visuospatial memory*** | | | | | | |
| Gestational age (weeks) | -0.104 | -1.024 – 0.816 | 0.821 | -0.518 | -1.595 – 0.559 | 0.346 |
| Surgical approach ^α^ | -2.189 | -7.380 – 3.002 | 0.402 | -1.951 | -7.352 – 3.450 | 0.479 |
| Duration of anaesthetic exposure (minutes) | -0.003 | -0.010 – 0.004 | 0.365 | -0.003 | -0.012 – 0.006 | 0.472 |
| Duration of intubation ^β^ | 0.178 | -5.799 – 6.155 | 0.953 | 0.949 | -5.132 – 7.030 | 0.760 |
| SES ^γ^ | 3.546 | -1.963 – 9.054 | 0.202 | 3.212 | -2.213 – 8.636 | 0.246 |
| *Potential predictors for impaired* ***delayed visuospatial memory*** | | | | | | |
| Gestational age (weeks) | -0.008 | -0.579 – 0.563 | 0.978 | -0.476 | -1.126 – 0.175 | 0.151 |
| Surgical approach ^α^ | -0.730 | -4.008 – 2.548 | 0.657 | -0.365 | -3.487 – 2.757 | 0.819 |
| Duration of anaesthetic exposure (minutes) | -0.002 | -0.006 – 0.002 | 0.289 | 0.000 | -0.005 – 0.006 | 0.905 |
| Duration of intubation ^β^ | -2.822 | -6.151 – 0.507 | 0.095 | -3.352 | -7.119 – 0.414 | 0.081 |
| SES ^γ^ | 4.359 | 1.110 – 7.607 | **0.010** | 4.471 | 1.409 – 7.532 | **0.004** |
| *Potential predictors for impaired* ***planning*** | | | | | | |
| Gestational age (weeks) | -0.082 | -0.404 – 0.239 | 0.608 | -0.166 | -0.550 – 0.218 | 0.397 |
| Surgical approach ^α^ | -0.340 | -2.043 – 1.364 | 0.689 | -0.501 | -2.263 – 1.262 | 0.578 |
| Duration of anaesthetic exposure (minutes) | -0.001 | -0.004 – 0.002 | 0.470 | -0.002 | -0.005 – 0.000 | 0.104 |
| Duration of intubation ^β^ | 0.728 | -1.246 – 2.701 | 0.460 | 1.851 | -0.430 – 4.131 | 0.112 |
| SES ^γ^ | -1.022 | -2.899 – 0.854 | 0.277 | -1.090 | -2.782 – 0.603 | 0.207 |
